# The generalized Sellmeier equation for air

**DOI:** 10.1038/srep46111

**Published:** 2017-08-24

**Authors:** A. A. Voronin, A. M. Zheltikov

**Affiliations:** 1 Physics Department, International Laser Center, M. V. Lomonosov Moscow State University, Moscow 119992, Russia; 2 Department of Physics and Astronomy, Texas A&M University, College Station, TX, 77843, USA; 3 Russian Quantum Center, Skolkovo, Moscow Region, 143025, Russia; 4 Kazan Quantum Center, A.N. Tupolev Kazan National Research Technical University, 420126 Kazan, Russia

## Abstract

We present a compact, uniform generalized Sellmeier-equation (GSE) description of air refraction and its dispersion that remains highly accurate within an ultrabroad spectral range from the ultraviolet to the long-wavelength infrared. While the standard Sellmeier equation (SSE) for atmospheric air is not intended for the description of air refractivity in the mid-infrared and long-wavelength infrared, failing beyond, roughly 2.5 μm, our generalization of this equation is shown to agree remarkably well with full-scale air-refractivity calculations involving over half a million atmospheric absorption lines, providing a highly accurate description of air refractivity in the range of wavelengths from 0.3 to 13 μm. With its validity range being substantially broader than the applicability range of the SSE and its accuracy being at least an order of magnitude higher than the accuracy that the SSE can provide even within its validity range, the GSE-based approach offers a powerful analytical tool for the rapidly progressing mid- and long-wavelength-infrared optics of the atmosphere.

Atmospheric optics is one of the earliest fields in all of the natural sciences^1^. Over many centuries, its primary focus has been on the visible range, where the colors of the sky, optical atmospheric phenomena, and the light from astronomical objects could be detected and studied either by direct visual observation or with the help of magnifying optics^2^. In the modern age of high-precision optical instruments and advanced photonic technologies, the needs of observational astronomy, as well as laser range finding, guidance, navigation, and remote sensing are still largely met in the visible and near-infrared ranges^3^, where atmospheric air is highly transparent to electromagnetic radiation. Yet, as one of the major trends in the area, the progress in mid-infrared technologies^4^ pushes the frontiers of atmospheric optics, calling for a detailed quantitative understanding of optical properties of the atmosphere in the mid- and long-wavelength infrared. Recently developed laser sources of high-peak-power ultrashort pulses in the mid-infrared^5^,^6^ offer a unique tool for atmospheric sciences. Such laser sources have been shown to enable the generation of mid-infrared laser filaments in the atmosphere^6^,^7^, opening the routes toward new regimes of long-distance signal transmission and remote sensing of the atmosphere^8^.

To fully unleash the potential of this new emerging technology, a deeper understanding of the group-velocity dispersion (GVD) of atmospheric air is needed. This call includes a quest for anomalous-GVD ranges where dispersion-induced stretching of ultrashort pulses could be suppressed and soliton transmission of powerful electromagnetic signal would be possible. However, because of a complex behavior of the refractive index of air, *n*(*ω*), within molecular absorption bands and in the wings of these bands, such an analysis is difficult both conceptually and technically. Full *n*(*ω*) calculations using the high-resolution transmission molecular absorption (HITRAN) database of infrared line transitions^9^ are both time- and labor-consuming, motivating a search for a compact closed-form description of atmospheric refractivity that would enable an approximate, yet accurate analysis of not only the refractive index, but also the GVD of atmospheric air, as well as its higher order dispersion parameters.

In the visible and near-infrared range, where atmospheric air is highly transparent, the dispersion of *n*(*ω*) is conveniently described in terms of a closed-form Sellmeier equation, whose phenomenologic coefficients have been defined with a very high accuracy^10^,^11^,^12^,^13^,^14^,^15^,^16^. In the mid-infrared (mid-IR) and long-wavelength-infrared (LWIR) ranges, however, the windows of high transparency of the atmosphere alternate with absorption bands related to rovibrational transitions of molecules, which give rise to complicated, rapidly oscillating frequency dependences of the refractive index. The standard, two- or three-term Sellmeier equation for atmospheric air fails when extended beyond its applicability range and applied to the description of *n*(*ω*) in the mid-IR and LWIR ranges, where molecular absorption bands start to play a significant role. The question as to whether or not a closed-form extension of the Sellmeier equation to these ranges is possible remains open.

Here, we address this question by presenting a generalized Sellmeier equation for air in atmospheric transparency regions, which provides a uniform, highly accurate description of air refraction and its dispersion within an ultrabroad spectral range from the ultraviolet (UV) to the LWIR. When extended to include a few most important rovibrational molecular bands along with electronic absorption bands in the ultraviolet, this approximation is shown to agree remarkably well with full-scale air-refractivity calculations based on the most comprehensive databases comprising over half a million atmospheric absorption lines. Staying accurate typically within 10^−10^ in the wavelength range spanning at least from 0.3 to 13 μm, this approximation provides a powerful analytical tool for the rapidly progressing mid- and long-wavelength-infrared optics of the atmosphere.

## The generalized Sellmeier equation for air

### Basic equations

We start with a generic model of air refractivity treating the optical response of air in terms of a superposition of independent oscillators:


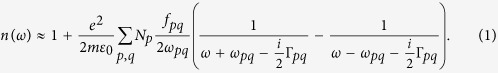


Here, *N*_*p*_ is the density of molecules or atoms of sort *p*, *ω*_*pq*_*, Γ*_*pq*_, and *f*_*pq*_ are the frequency, the linewidth, and the oscillator strength of the *q*th resonance in the spectrum of molecules or atoms of sort *p*, *m*, and *e* are the electron mass and charge, and *ε*_0_ is the dielectric permittivity of vacuum.

**Figure 1:**
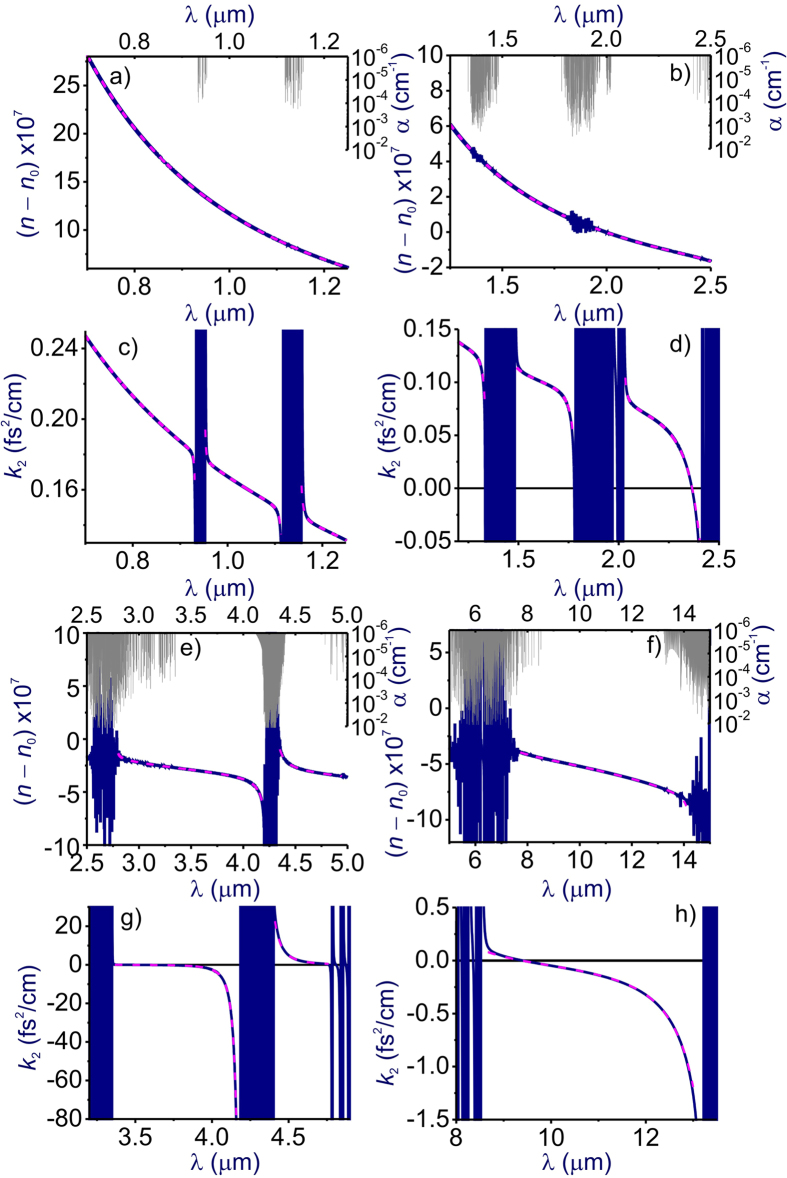
The refractive index (**a**–**d**) and the group-velocity dispersion (**e**–**h**) calculated as functions of the wavelength using the full model of air refractivity based on Eq. (1) including the HITRAN-database manifold of atomic and molecular transitions (blue line) and Eq. (5) including *M* = 15 terms with parameters as specified in Table 1 (pink line): (**a**,**e**) visible and near-IR, (**b**,**f**) near-IR, (**c**,**g**) mid-IR, and (**d**,**h**) mid-IR and LWIR ranges. Absorption spectrum of air is shown by grey shading. Atmospheric air is modeled as a mixture of molecular and atomic gases with densities *N*_N2_ = 1.987 ∙ 10^19^ cm^−3^, *N*_O2_ = 5.3291 ∙ 10^18^ cm^−3^, *N*_Ar_ = 2.3763 ∙ 10^17^ cm^−3^, *N*_H2O_ = 7.0733 ∙ 10^16^ cm^−3^ (10% humidity), and *N*_CO2_ = 9.4136 ∙ 10^15^ cm^−3^. The temperature is 296 K, *n*_0_ = 1.000273.

**Figure 2:**
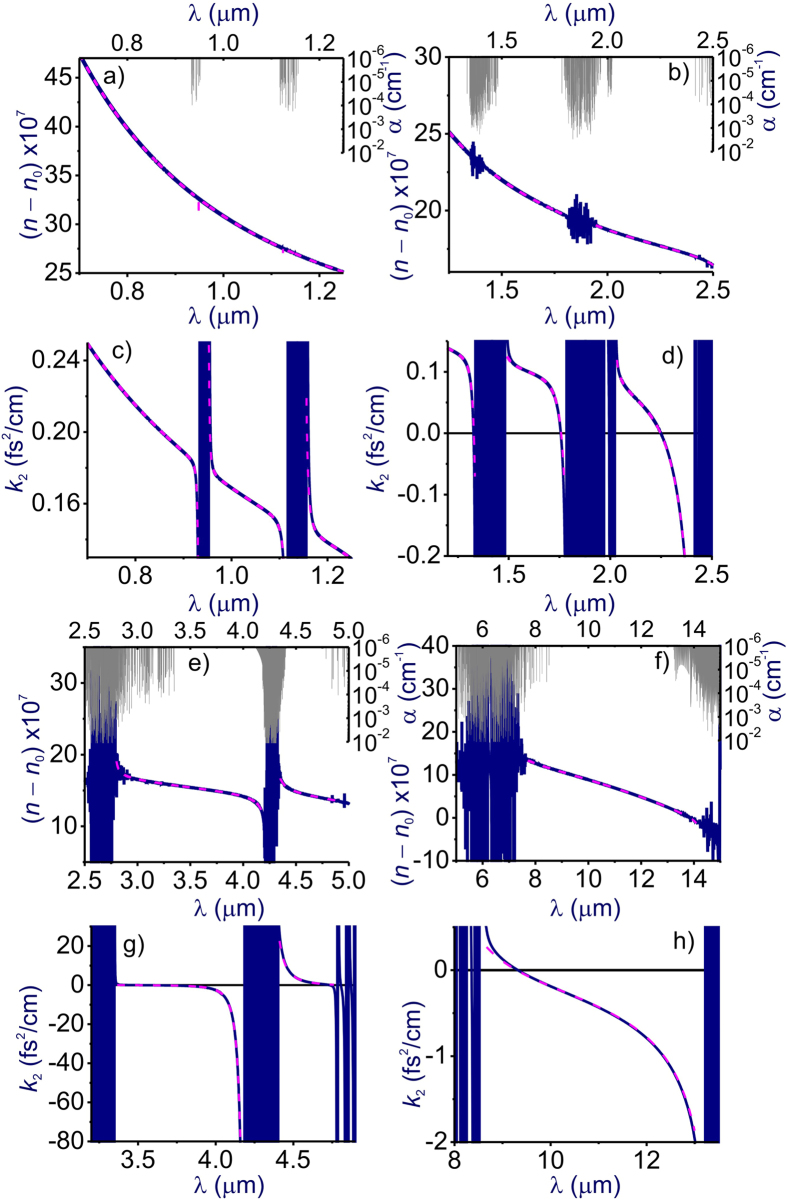
The same as in Fig. 1 for *N*_H2O_ = 2.8305 ∙ 10^17^ cm^−3^ (40% humidity).

With constants *ω*_*pq*_*, Γ*_*pq*_, and *f*_*pq*_ taken from the HITRAN database^9^, Eq. (1) provides ultrahigh-accuracy predictions for the refractive index of air within the UV, visible, mid-IR, and LWIR ranges. However, each such calculation for given humidity and fixed densities of air constituents, referred to hereinafter as full-model analysis (FMA), involves a total of more than 650,000 sets of the *ω*_*pq*_*, Γ*_*pq*_, and *f*_*pq*_ constants for N_2_, O_2_, H_2_O, CO_2_, O_3_, CH_4_, CO, Ar, and Ne and returns *n*(*ω*) profiles (Figs 1 and 2) that wildly oscillate within molecular absorption bands, offering little promise as an analytical tool, e.g., for the identification of GVD anomalies.

Our main goal here is to provide a compact closed-form description of atmospheric refractivity that would enable an approximate, yet accurate analysis of the refractive index of atmospheric air, as well as its GVD and higher order dispersion within atmospheric transparency regions in the spectral range stretching from the UV to the LWIR. To this end, we resort to the Sellmeier equation, which has long been in use as a tool for an approximate description of the dispersion of optical materials within their transparency regions.

For atmospheric air, the standard Sellmeier equation (SSE) is usually presented as^11^,^12^,^13^,^14^,^15^,^16^


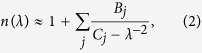


where *λ* is the wavelength and *B*_*j*_ and *C*_*j*_ are phenomenologically defined constants.

The Sellmeier equation of the form of Eq. (2) including only two UV resonant terms with *B*_1_ = 0.05792105 μm^−2^, *B*_2_ = 0.00167917 μm^−2^, *C*_1_ = 238.0185 μm^−2^, and *C*_2_ = 57.362 μm^−2^ is known to provide a highly accurate description of the refractive index of atmospheric air^11^,^12^,^13^,^14^,^15^. This equation with temperature- and humidity-dependent coefficients has been generally accepted and approved (e.g., by the Joint Commission for Spectroscopy, the Advisory Committee for the Definition of Metre, and the Commission of the International Astronomical Union) as a standard for high-precision spectroscopy and interferometry in atmospheric air, as well as for geodetic surveying. While the accuracy of refractive index calculations within parts in 10^−8^ has been achieved by applying Eq. (2) within the range of wavelengths from approximately 200 nm to 1 μm already some six decades ago^12^, most recent corrections to the *B*_*j*_ and *C*_*j*_ coefficients enable an even more accurate analysis of atmospheric refractivity in this spectral region^16^.

**Figure 3:**
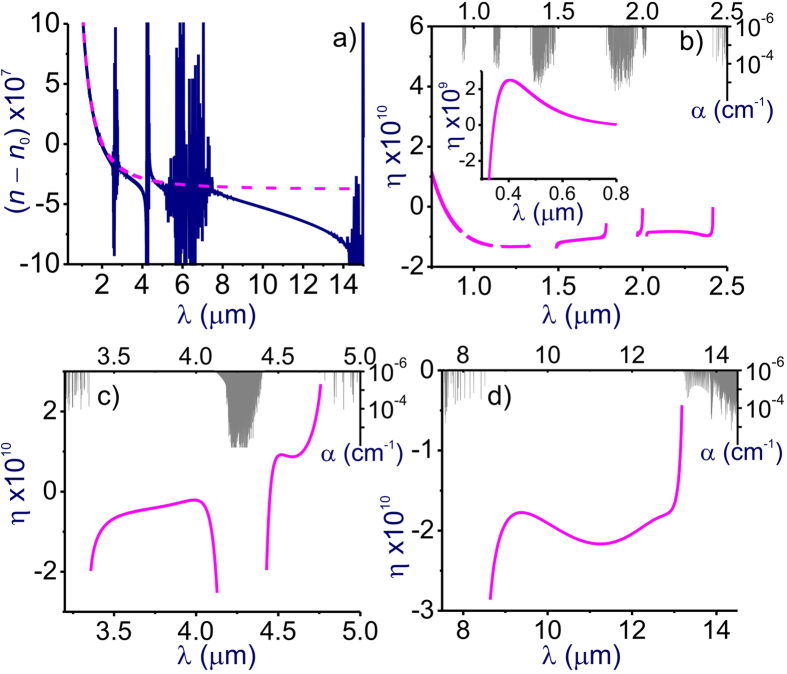
(**a**) The refractive index calculated as a function of the wavelength using the full model of air refractivity based on Eq. (1) including the HITRAN-database manifold of atomic and molecular transitions (blue solid line) and the standard two-term Sellmeier equation [Eq. (2)] for air with parameters *B*_*j*_ and *C*_*j*_ as specified in the text (pink dashed line). (**b**–**d**) The approximation error *η* as a function of the wavelength in the near-IR (**b**), mid-IR (**c**), and LWIR (**d**) ranges. Parameters of calculations Eq. (1) are as specified in Fig. 1. The absorption spectrum of air is shown by grey shading.

The SSE description, however, fails within the spectral region where the rovibrational transitions of molecular constituents of atmospheric air become important – most notably, in the mid-IR and LWIR ranges. To illustrate this argument, the refractive index of air calculated with the full model of Eq. (1) is compared in Fig. 3a with the results of calculations performed with the standard, two-term (*j* = 1, 2) version of Eq. (2). While in the 0.2–1.7 μm wavelength range, the SSE of Eq. (2) provides a very accurate fit for *n*(*λ*), prominent molecular absorption bands in the mid-IR and LWIR lead to a dramatic discrepancy between SSE and FMA predictions for *λ* > 1.7 μm.

In search for a more adequate, yet compact model for *n*(*ω*) within the transparency range, we simplify Eq. (1) taking into account that *Γ*_*pq*_ 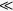 |*ω*_*pq*_ − *ω*| outside absorption bands and *Γ*_*pq*_ 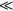 *ω*_*pq*_ + *ω* within the entire parameter space of interest,


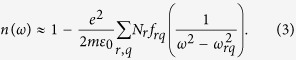


Here, instead of summing over the sort *p* of molecular and atomic species, we sum over the absorption bands, enumerated by *r*, while the index *q* still runs through all the individual lines within the *r*th absorption band (e.g., individual rovibrational lines in the case of molecular absorption bands).

As a next step, we approximate the sum over individual transitions in each term *r* in Eq. (3) as


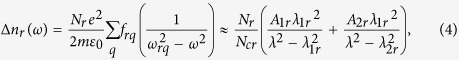


where *N*_*cr*_ = *mε*_0_*ω*^2^/*e*^2^ is the critical plasma density, *A*_1*r*_ and *A*_2*r*_ are the coefficients such that *A*_1*r*_ + *A*_2*r*_ = 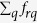/2, and *λ*_1*r*_ and *λ*_2*r*_are characteristic wavelengths, chosen as best-fit parameters.

Eq. (4) leads to the following generalized Sellmeier equation (GSE) for the refractive index of air:


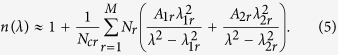


With *e*, *m*, and *ω* combined into *N*_cr_ in Eqs (4) and (5), it is straightforward to see that our dispersion relation is fully consistent with the universal high-frequency limit for the dielectric function, as it recovers the expression for the refractive index of a free-electron gas in the *ω* ≫ *ω*_*pq*_*, Γ*_*pq*_ limit, 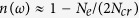. This dictates the normalization of the oscillator strengths *f*_*pq*_ in Eq. (1)^17^, as well as the constants *A*_*jr*_ in Eqs (4) and (5).

When represented as Eq. (5), the closed-form expression for air refractivity is instrumental for the analysis of air GVD, 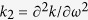. Indeed, differentiation of Eq. (5) leads to


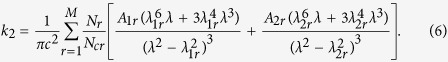


It is straightforward to see now that, in agreement with a generic behavior of a harmonic-oscillator response, each molecular rovibrational mode *r* gives rise to anomalous GVD in the high-frequency wing of its absorption band, i.e., for *λ* < *λ*_1*r*_, *λ*_2*r*_. Whether or not the net GVD is anomalous in the high-frequency tail of an *r*th molecular band depends on how intense this molecular mode is, that is, how large *A*_1*r*_ and *A*_2*r*_ are, compared to the other terms in the sum over *r* in Eqs (5) and (6).

### Physical assignment

In Figs 1 and 2, we compare the refractive index *n*(*λ*) and the GVD *k*_2_(*λ*) of atmospheric air calculated using the GSE approximation of Eqs (5) and (6) with FMA calculations of *n*(*λ*) and *k*_2_(*λ*) including more than 650,000 atomic and molecular transitions from the HITRAN database. Atmospheric air is modeled in these calculations as a mixture of molecular and atomic gases with densities *N*_N2_ = 1.987 ∙ 10^19^ cm^−3^, *N*_O2_ = 5.3291 ∙ 10^18^ cm^−3^, *N*_Ar_ = 2.3763 ∙ 10^17^ cm^−3^, and *N*_CO2_ = 9.4136 ∙ 10^15^ cm^−3^. The density of water molecules, *N*_H2O_, is calculated as


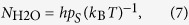


where *T* is the air temperature, *h* is the humidity, *k*_B_ is the Boltzmann constant, and *p*_S_ is the saturated vapor pressure, defined as^18^ *p*_S_ = *p*_c_ exp[*τ*(*α*_1_*θ* + *α*_2_*θ*^1.5^ + *α*_3_*θ*^3^ + *α*_4_*θ*^3.5^ + *α*_5_*θ*^4^ + *α*_6_*θ*^7.5^)], with *τ* = *T*_c_/*T*, *θ* = 1−*T*/*T*_c_, *T*_c_ ≈ 647.096 K being the critical-point temperature of water, *p*_c_ ≈ 22.064 MPa being the critical-point pressure of water, and *α*_1_ ≈ −7.85951783, *α*_2_ ≈ 1.84408259, *α*_3_ ≈ −11.7866497, *α*_4_ ≈ 22.6807411, *α*_5_ ≈ −15.9618719, *α*_6_ ≈ 1.80122502^19^.

**Figure 4:**
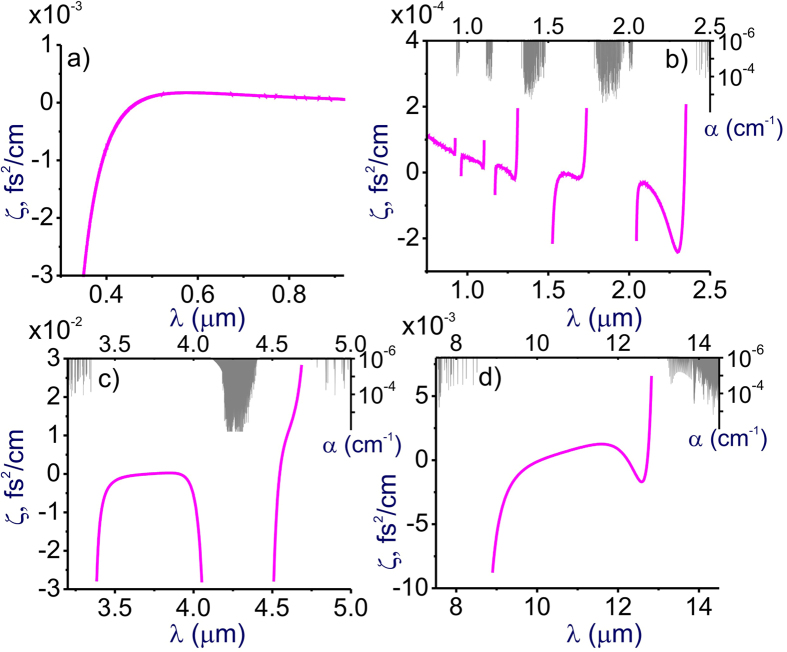
The GVD approximation error *ζ* as a function of the wavelength in the visible (**a**), near-IR (**b**), mid-IR (**c**), and LWIR (**d**) ranges. Parameters of calculations Eq. (1) are as specified in Fig. 1. The absorption spectrum of air is shown by grey shading.

**Figure 5:**
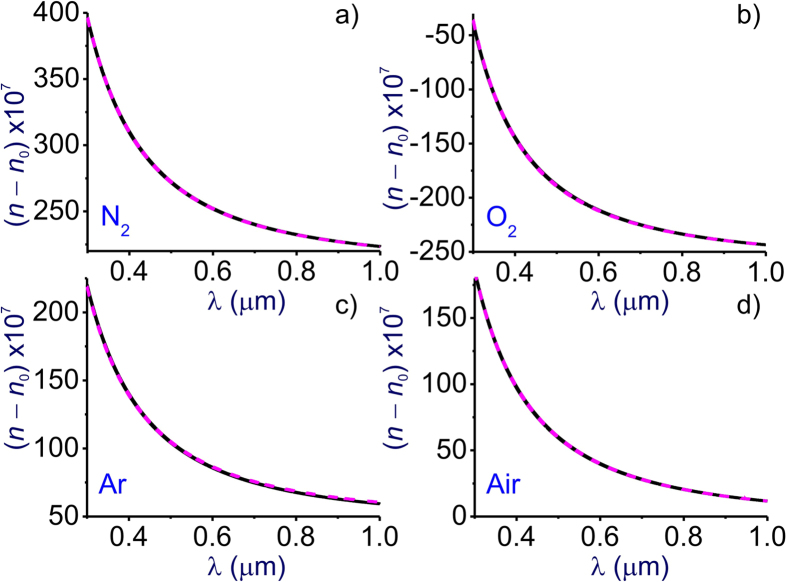
The refractive index calculated as a function of the wavelength using (blue solid line) the standard Sellmeier equation for N_2_ (**a**), O_2_ (**b**), Ar (**c**), and [Eq. (2)] for air (**d**) and (pink dashed line) Eq. (5) including only the terms with *r* = 12, 13, 14, 15 with parameters as specified in Table 1 for (**a**) *N*_N2_ = 2.688 ∙ 10^19^ cm^−3^, (**b**) *N*_O2_ = 2.504 ∙ 10^19^ cm^−3^, (**c**) *N*_Ar_ = 2.879 ∙ 10^19^ cm^−3^, and (**d**) the standard gas content of atmospheric air with parameters as specified in Fig. 1.

**Figure 6:**
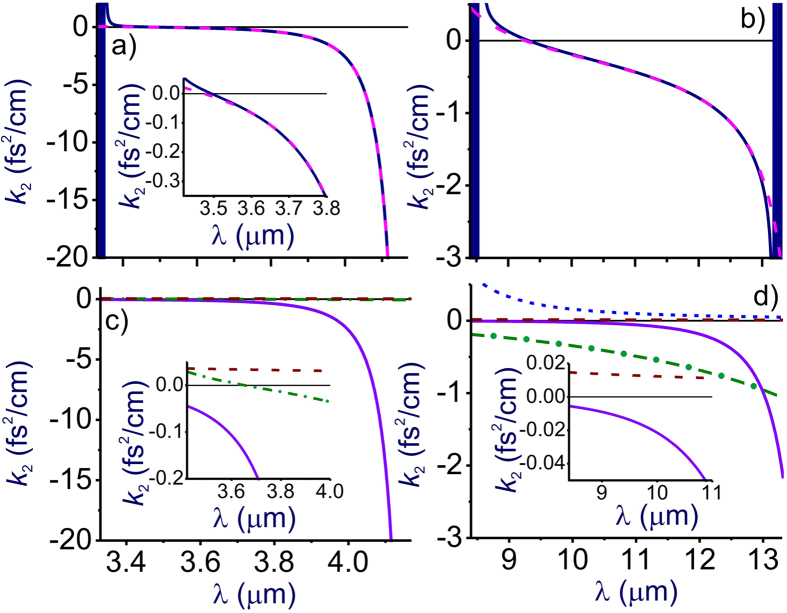
(**a**,**b**) Group velocity dispersion *k*_2_ calculated as a function of the wavelength using the full model of air refractivity based on Eq. (1) including the HITRAN-database manifold of atomic and molecular transitions (blue solid line) and Eq. (6) including only the terms with (**a**) *r* = 2, 6, 7, 12, 13 and (**b**) *r* = 1, 5, 6, 12, and 13 with parameters as specified in Table 1 (pink dashed line). (**c**,**d**) The GVD contributed by individual terms and group of terms in Eq. (6): (**c**) *r* = 2 CO_2_ term (solid line), combined contribution of H_2_O terms with *r* = 6 and 7 (dash–dotted line), and combined contribution of the N_2_ and О_2_ UV terms with *r* = 12 and 13 (dashed line); (**d**) *r* = 1 CO_2_ term (solid line), *r* = 5 H_2_O term (dash–dotted line), *r* = 6 H_2_O term (dotted line), and combined contribution of the N_2_ and О_2_ UV terms with *r* = 12 and 13 (dashed line). The insets show the close-up view of the GVD profile near the zero-GVD wavelength. Parameters of calculations are as specified in Fig. 2.

Comparison of these GSE and FMA calculations shows (see also Figs 3, 4, 5, 6) that the entire *n*(*λ*) profile of atmospheric air within the range of wavelengths from at least 0.3 to 13 μm can be accurately approximated with the sum in *r* in Eq. (5) extended over *M* = 15 most significant molecular and atomic absorption bands of atmospheric air. These bands, listed in Table 1 (also shown by grey shading in Figs 1 and 2), include four most intense rovibrational bands of CO_2_ molecules in the near-IR, mid-IR, and LWIR ranges (*r* = 1–4), one terahertz (THz) absorption band of H_2_O (*r* = 5), six near-to-long-wavelength-IR absorption bands of H_2_O (*r* = 6–11), as well as four UV bands comprising electronic transitions of N_2_^20^ (*r* = 12), O_2_^20^ (*r* = 13), Ar^21^,^22^,^23^ (*r* = 14), and H_2_O^20^ (*r* = 15).

Along with a quite natural set of molecular modes in the near-IR, mid-IR, and LWIR ranges, our GSE model includes, as a much less obvious element, the terahertz 000 – 000 mode of H_2_O (*r* = 5). As the absorption band corresponding to this molecular mode peaks at around 43 μm, i.e., a frequency of about 7 THz, the motives behind the inclusion of this term into the GSE are far from being evident. In fact, the question as to whether or not this terahertz molecular mode (as well as any other far-IR or terahertz mode) can manifest itself in any detectable way in the LWIR range is addressed in our approach through an accurate quantitative assessment of the effect that this mode has on the refractive index of air and its GVD through Eqs (5) and (6). Our analysis presented below in this paper shows that, due to the remarkable intensity of spectral lines within this band, inducing a very strong absorption of air, the *r* = 5 terahertz mode of H_2_O plays a very significant role in the dispersion of air in the LWIR atmospheric transparency window and, therefore, has to be included in the GSE model.

In the case of simple parallel rovibrational modes of molecules, the two terms appearing in Eqs (4)–(6) have a clear physical meaning as they are identified with the contributions of the P and R branches of rovibrational transitions^17^, corresponding, respectively, to Δ*J* = −1 and Δ*J* = +1 selection rules in the rotational quantum number *J*. The wavelengths *λ*_1*r*_ and *λ*_2*r*_ are then understood as the central wavelengths of these two branches. For more complicated rovibrational modes of molecules, as well as for UV absorption bands, related to electronic transitions in atoms and molecules (*r* = 12–15 in Table 1), the two-term structure of Eq. (4) does not have such a clear physical assignment, but still makes sense in practical terms. With the wavelengths *λ*_1*r*_ and *λ*_2*r*_ chosen near the high- and low-frequency edges of each such band, the accuracy of such a two-term approximation of Δ*n*_*r*_ is always much more accurate than the accuracy attainable with a single term in Δ*n*_*r*_.

### Approximation accuracy

To quantify the accuracy of the GSE approximation of Eq. (5), we introduce the approximation errors *η* = *n*_GSE_ − *n*_FMA_ and *ζ* = (*k*_2_)_GSE_ − (*k*_2_)_FMA_ where *n*_GSE_ and (*k*_2_)_GSE_ are the refractive index and the GVD calculated with the use of the GSE of Eq. (5) and *n*_FMA_ and (*k*_2_)_FMA_ are the refractive index and the GVD calculated with the FMA of Eq. (1) including the entire manifold of a total of about 650,000 HITRAN-database atomic and molecular transitions in atmospheric air. In Figs 3 and 4, the approximation errors *η* and *ζ* are plotted as functions of the wavelength.

When extended to include a sum over *M* = 15 terms with parameters as specified in Table 1, the GSE approximation of Eq. (5) is seen to agree remarkably well with FMA air-refractivity calculations (see also Figs 1 and 2), with the approximation error |*η*| kept at the level of 10^−10^ almost everywhere within the transparency region of atmospheric air in the range of wavelengths from 0.3 to 13 μm (Fig. 3b–d). Only within very narrow spectral regions near the strongest molecular bands in the mid-IR (Fig. 3c) and the LWIR (Fig. 3d), the approximation error |*η*| becomes larger than 10^−10^. Moreover, within a spectroscopically significant spectral range from approximately 3.4 to 4.1 μm, GSE predictions deviate from FMA calculations by |*η*| < 10^−10^. Remarkably, not only does the GSE provide a much broader, 0.3–13 μm validity range compared to the SSE, but also the accuracy of the GSE model of air refractivity, as can be seen from Fig. 3a–d, is at least an order of magnitude higher than the accuracy that the SSE provides within its much narrower applicability range.

Since the GVD *k*_2_ is controlled by the second-order derivative of *k*(*λ*), it is much more sensitive to small variations in the refractive index. As a result, even very weak molecular bands that lead to almost no absorption and that are almost invisible in *n*(*λ*) dependences tend to show up as prominent features in *k*_2_(*λ*) profiles (Figs 1 and 2), which inevitably translate into larger GVD approximation errors *ζ* in narrow spectral regions near the edges of these bands (Fig. 4b–d). Still, within the atmospheric transmission range in between these molecular bands, the GSE approximation provides a high accuracy of GVD calculations (Figs 1, 2 and 6a,b). For *λ* < 4.1 μm, the GVD approximation error |*ζ*| is seen to exceed 1 fs^2^/cm only in the immediate neighborhood of molecular bands (Fig. 4b,c), staying typically within 1–2 fs^2^/cm within most of the LWIR atmospheric transmission window (Fig. 4d).

## Uniform description of air refraction: from the UV to the LWIR

### Visible and near-infrared

As can be seen from Fig. 1a,b,e and f, within the main, visible-to-near-IR atmospheric transmission window, all the way up to approximately 1.8 μm, weak absorption bands of H_2_O (*r* = 9–11, Table 1) have almost no influence on the behavior of *n*(*λ*), but show up in the GVD of atmospheric air. These molecular modes are manifested as regions of rapidly oscillating *k*_2_(*λ*), centered at approximately 0.94, 1.14, and 1.40 μm, with narrowband anomalous GVD, *k*_2_ < 0, in the high-frequency outskirts of these bands (Fig. 1a,b,e,f). The bandwidths of these GVD anomalies are not sufficient to enable the generation of femtosecond solitons, but could potentially help compensate group-delay effects in multicolor standoff detection and remote sensing schemes using ultrashort laser pulses.

Both *n*(*λ*) and *k*_2_(*λ*) profiles are accurately described within this wavelength range by the standard, two-term Sellmeier equation [Eq. (2)]. Instead of the two terms of the SSE for air, where phenomenologically defined *B*_*j*_ and *C*_*j*_ parameters (*j* = 1, 2) are assigned the values as specified above, our GSE model of Eq. (5) includes four UV terms (*r* = 12–15, Table 1) each assigned to a specific, though sometimes complicated manifold of electronic transitions in one of the atmospheric constituent gases – N_2_ (*r* = 12), O_2_ (*r* = 13), Ar (*r* = 14), and H_2_O (*r* = 15). With such an assignment, we aim to unveil the physical meaning behind the highly accurate, but still phenomenological two-term SSE for atmospheric air, as well as to sustain the physically meaningful uniformity over all the terms in Eqs (5) and (6).

In Fig. 5a–d, we plot the refractive index calculated as a function of the wavelength using the SSE for the individual atmospheric constituent gases providing most significant UV resonances (N_2_, O_2_, and Ar, Fig. 5a–c) and air (Fig. 5d) versus the *n*(*λ*) calculated with the GSE model of Eq. (5) including only the UV terms (i.e., terms with *r* = 12–15) with parameters as specified in Table 1. The SSE for air is defined here as two-term Eq. (2) with parameters *B*_*j*_ and *C*_*j*_ as specified above. In the case of N_2_, O_2_, and Ar, we follow the tradition^24^,^25^,^26^,^27^ by taking the dispersion equation in the form of Eq. (2) with the *j* = 2 term replaced with a constant, *D*, and by using a standard set of parameters: *B*_1_ = 3.243157 ∙ 10^−2^, *C*_1_ = 144.0 μm^−2^, and *D* = 6.8552 ∙ 10^−5^ for N_2_^24^, *B*_1_ = 9.708931 ∙ 10^−3^, *C*_1_ = 75.4 μm^−2^, and *D* = 1.181494 ∙ 10^−4^ for O_2_^25^,^26^, and *B*_1_ = 3.0182943 ∙ 10^−2^, *C*_1_ = 144.0 μm^−2^, and *D* = 6.7867 ∙ 10^−5^ for Ar^27^. Comparison of calculations presented in Fig. 5a–c shows that each of the N_2_, O_2_, and Ar UV terms (*r* = 12, 13, and 14) in Eq. (5) provides a highly accurate description of the refraction induced by each of these atmospheric constituents in the UV, visible, and near-IR range, where infrared molecular absorption is still of no significance. Calculations presented in Fig. 4d show that the GSE model (pink dashed line in Fig. 5d) agrees very well with the predictions of the two-term SSE for atmospheric air (blue solid line in Fig. 5d).

Identifying a clear physical assignment of each term in the GSE model of Eqs (5) and (6) with regard to specific absorption bands of atmospheric constituent gases is, of course, much more than a matter of pure scientific satisfaction. With the relation of the individual terms in Eqs (5) and (6) to a specific atmospheric constituent gas established, these equations provide a powerful analytical tool to study the refractive index of air and its GVD as functions of the air humidity, temperature, and the partial densities of atmospheric constituents. Results of such studies are illustrated in Figs 1 and 2, where the *n*(*λ*) and *k*_2_(*λ*) profiles are calculated for different levels of air humidity. As can be seen from these calculations, the increase of water content in atmospheric air enhances all the H_2_O-related features in *n*(*λ*) and *k*_2_(*λ*), including GVD anomalies induced by H_2_O bands in the mid-IR and LWIR ranges (see also Fig. 6).

### Mid-infrared

Further into the near-IR and especially in the mid-IR range, molecular absorption bands become a much more prominent factor, giving rise to prominent features not only in *k*_2_(*λ*), but also in *n*(*λ*) profiles (Fig. 1b,c,f,g). Specifically, partially overlapping absorption bands of CO_2_ (*r* = 3) and H_2_O (*r* = 7), as well as the asymmetric-stretch rovibrational band of CO_2_ (*r* = 2) show up in this spectral range as the regions of fast oscillating *n*(*λ*) and *k*_2_(*λ*) centered at around 2.7 and 4.3 μm, respectively (Fig. 1c,g). GVD anomalies with *k*_2_ < 0, observed in the high-frequency neighborhood of these absorption bands, are now broadband enough to support femtosecond soliton transients, subject to high-order dispersion, which becomes especially strong toward the edges of the atmospheric transparency windows at approximately 2.45 and 4.2 μm (Fig. 6a).

Of particular interest is the mid-IR GVD anomaly that occurs in the atmospheric transparency region right outside the asymmetric-stretch rovibrational band of CO_2_ and that covers the range of wavelengths from approximately 3.5 to 4.17 μm (Figs 1g and 6a,c). With high-peak-power sources of ultrashort pulses now available in this spectral range^5^,^6^,^7^,^8^, soliton transmission of powerful mid-IR signals should become possible. As can be seen from Fig. 6a,c, the GVD sum of Eq. (6) is dominated in this region by the negative *r* = 2 term provided by the asymmetric-stretch rovibrational mode of CO_2_. As the absolute value of this term, decreases for shorter wavelengths away from the asymmetric-stretch CO_2_ absorption band (solid line in Fig. 6c), the normal-GVD terms related to UV electronic transitions of N_2_ and O_2_ (*r* = 12, 13, dashed line in Fig. 6c) start to play a noticeable role in the net GVD, which eventually reverses its sign at *λ*_z_ ≈ 3.5 μm.

As can be seen from Figs 1 and 2, an increase in air humidity enhances all the H_2_O-related features in *n*(*λ*) and *k*_2_(*λ*). As one of the most drastic manifestations of a higher air humidity in the mid-IR range, we observe a drastic broadening of the range of anomalous GVD in the high-frequency neighborhood of the *r* = 3 CO_2_ band. For 10% air humidity, this GVD anomaly stretches from the edge of the CO_2_ absorption band to a zero-GVD wavelength *λ*_z_ ≈ 2.37 μm (Fig. 1d). An increase in air humidity to 40% is seen to shift the zero-GVD point of this dispersion anomaly to *λ*_z_ ≈ 2.25 μm (Fig. 2d).

### Long-wavelength infrared

In the LWIR range, the atmospheric transparency window, covering the spectral region from approximately 7.7 to 13.5 μm, is bounded by strong absorption bands of H_2_O (*r* = 6) and CO_2_ (*r* = 1), centered at, roughly, 6.2 and 14.6 μm, respectively (Fig. 1d,h). As, perhaps, the most striking finding, we observe a broadband GVD anomaly within this range, stretching from *λ*_z_ ≈ 9.3 μm all the way down to the edge of the CO_2_ absorption band at around 13.5 μm (Fig. 1h). Unlike the 3.5–4.2 μm mid-IR GVD anomaly, which is dominated by the anomalous GVD provided solely by the asymmetric-stretch rovibrational mode of CO_2_ (Fig. 6a,c), this 9.3–13.5 μm LWIR GVD anomaly is more complicated, with several molecular bands having a significant influence on the behavior of *k*_2_(*λ*) in this wavelength range (Fig. 6b,d).

In the atmospheric transparency region right next to the edge of the CO_2_ absorption band, that is, for *λ* > 13 μm, the GVD is, of course, dominated by the *r* = 1 CO_2_ molecular mode (solid line in Fig. 6d). For *λ* < 13 μm, however, the strong terahertz absorption band of H_2_O (*r* = 5) comes into play, providing, perhaps surprisingly, an even more significant contribution to the anomalous GVD (dash-dotted line in Fig. 6d). As a striking manifestation of this terahertz mode of H_2_O, we observe a drastic increase in the magnitude of the GVD anomaly in the atmospheric transparency region near the *r* = 1 CO_2_ band. With air humidity increased from 10% to 40%, the GVD at *λ* = 12 μm is seen to change from *k*_2_ = −0.35 fs^2^/cm in Fig. 1h to *k*_2_ = −0.82 fs^2^/cm in Fig. 2h.

To make things even more complicated, the *r* = 6 absorption band of H_2_O, which is much weaker than the *r* = 5 terahertz H_2_O absorption band, but which lies in the immediate neighborhood of the spectral region of interest, also has its part to play in the behavior of *k*_2_(*λ*) in the LWIR atmospheric transmission window. Along with UV electronic transitions of N_2_ and O_2_ (*r* = 12, 13, dashed line in Fig. 6d), this molecular mode provides a positive GVD term in Eq. (6), which increases in its magnitude as the wavelength approaches its low-frequency edge at approximately 7.7 μm. The interplay of these factors defines the sign and the profile of *k*_2_(*λ*) in the LWIR atmospheric transmission window and controls the zero-GVD point, which occurs at *λ*_z_ ≈ 9.3 μm (Fig. 6b,d).

## Conclusion

To summarize, we presented a generalized Sellmeier equation, providing a uniform, highly accurate description of air refraction and its dispersion in atmospheric transmission windows within an ultrabroad spectral range spanning from the UV to the LWIR range. When extended to include a few most important rovibrational molecular bands along with electronic absorption bands in the ultraviolet, this approximation is shown to agree remarkably well with full-scale air-refractivity calculations based on the most comprehensive databases comprising over half a million atmospheric absorption lines. Remarkably, not only does the GSE provide a much broader, 0.3–13 μm validity range compared to the SSE, but also the accuracy of the GSE model of air refractivity is at least an order of magnitude higher than the accuracy that the SSE provides within its much narrower applicability range. Staying accurate typically within 10^−10^ in the wavelength range spanning at least from 0.3 to 13 μm, the GSE approximation provides a powerful analytical tool for the rapidly progressing mid- and long-wavelength-infrared optics of the atmosphere.

## Additional Information

**How to cite this article**: Voronin, A. A. and Zheltikov, A. M. The generalized Sellmeier equation for air. *Sci. Rep.* **7**, 46111; doi: 10.1038/srep46111 (2017).

**Publisher's note:** Springer Nature remains neutral with regard to jurisdictional claims in published maps and institutional affiliations.

## Figures and Tables

**Table 1:** Parameters of the generalized Sellmeier equation [Eq. (5)].

*r*	Mol/atom	Transitions	*A*_1*r*_, ps^2^	*A*_2*r*_, ps^2^	*λ*_1*r*_, nm	*λ*_2j_, nm	*N*_r_, 10^15^ cm^−3^
1	CO_2_	01^1^01–10^0^02 00^0^01–01^1^01 10^0^02–11^1^02 01^1^01–02^2^01 02^2^01–03^3^01 01^1^01–10^0^01 10^0^01–11^1^01	4.051 ∙ 10^−6^	1.010 ∙ 10^−6^	15131	14218	9.4136
2	CO_2_	00^0^01–00^0^11 01^1^01–01^1^11 02^2^01–02^2^11 10^0^02–10^0^12 10^0^01–10^0^11	2.897 ∙ 10^−5^	2.728 ∙ 10^−5^	4290.9	4223.1	
3	CO_2_	01^1^01–11^1^12 00^0^01–10^0^12 00^0^01–10^0^11 01^1^01–11^1^11	8.573 ∙ 10^−7^	6.620 ∙ 10^−7^	2684.9	2769.1	
4	CO_2_	00^0^01–20^0^12 00^0^01–20^0^11	1.550 ∙ 10^−8^	5.532 ∙ 10^−9^	2011.3	1964.6	
5	H_2_O	000–000	2.945 ∙ 10^−5^	6.583 ∙ 10^−8^	47862	16603	Eq. (7) at *T* = 296 K
6	H_2_O	000–010	3.273 ∙ 10^−6^	3.094 ∙ 10^−6^	6719.0	5729.9	
7	H_2_O	000–020 000–100 000–001	1.862 ∙ 10^−6^	2.788 ∙ 10^−6^	2775.6	2598.5	
8	H_2_O	000–011 000–110	2.544 ∙ 10^−7^	2.181 ∙ 10^−7^	1835.6	1904.8	
9	H_2_O	000–021 000–200 000–101	1.126 ∙ 10^−7^	2.336 ∙ 10^−7^	1417.6	1364.7	
10	H_2_O	000–111 000–210	6.856 ∙ 10^−9^	9.479 ∙ 10^−9^	1145.3	1123.2	
11	H_2_O	000–201	1.985 ∙ 10^−9^	2.882 ∙ 10^−9^	947.73	935.09	
12	N_2_	electronic transitions as specified in ref. 7	1.2029482	5.796725	85	24.546	19870
13	O_2_	electronic transitions^7^	0.26507582	7.734925	127	29.469	5329.1
14	Ar	electronic transitions^8^,^9^,^10^	0.93132145	7.217322	87	22.645	237.63
15	H_2_O	electronic transitions^7^	0.25787285	4.742131	128	34.924	Eq. (7) at *T* = 296 K

Notation: *v*_1_*v*_2_^*l*^*v*_3_*ξ* is a superposition of *v*_1_ symmetric-stretch, *v*_2_ bending, and *v*_3_ asymmetric-stretch vibrations with an angular momentum *l*, and Fermi-resonance perturbation parameter *ξ*.
